# The Loss-Function of the Male Sterile Gene *ZmMs33*/*ZmGPAT6* Results in Severely Oxidative Stress and Metabolic Disorder in Maize Anthers

**DOI:** 10.3390/cells11152318

**Published:** 2022-07-27

**Authors:** Ziwen Li, Shuangshuang Liu, Taotao Zhu, Xueli An, Xun Wei, Juan Zhang, Suowei Wu, Zhenying Dong, Yan Long, Xiangyuan Wan

**Affiliations:** 1 Shunde Graduate School, Zhongzhi International Institute of Agricultural Biosciences, Research Center of Biology and Agriculture, University of Science and Technology Beijing, Beijing 100024, China; liziwen@ustb.edu.cn (Z.L.); d202110465@xs.ustb.edu.cn (S.L.); b20170369@xs.ustb.edu.cn (T.Z.); xuelian@ustb.edu.cn (X.A.); weixun@ustb.edu.cn (X.W.); juanz@ustb.edu.cn (J.Z.); suoweiwu@ustb.edu.cn (S.W.); zydong@ustb.edu.cn (Z.D.); 2 Beijing Engineering Laboratory of Main Crop Bio-Tech Breeding, Beijing International Science and Technology Cooperation Base of Bio-Tech Breeding, Beijing Solidwill Sci-Tech Co., Ltd., Beijing 100192, China

**Keywords:** *ZmMs33*, SnRK1, reactive oxygen species, oxidative stress, metabolic reprogramming, anther and pollen development, male sterility, maize

## Abstract

In plants, oxidative stress and metabolic reprogramming frequently induce male sterility, however our knowledge of the underlying molecular mechanism is far from complete. Here, a maize genic male-sterility (GMS) mutant (*ms33-6038*) with a loss-of-function of the *ZmMs33* gene encoding glycerol-3-phosphate acyltransferase 6 (GPAT6) displayed severe deficiencies in the development of a four-layer anther wall and microspores and excessive reactive oxygen species (ROS) content in anthers. In *ms33-6038* anthers, transcriptome analysis identified thousands of differentially expressed genes that were functionally enriched in stress response and primary metabolism pathways. Further investigation revealed that 64 genes involved in ROS production, scavenging, and signaling were specifically changed in expression levels in *ms33-6038* anthers compared to the other five investigated GMS lines. The severe oxidative stress triggered premature tapetal autophagy and metabolic reprogramming mediated mainly by the activated SnRK1-bZIP pathway, as well as the TOR and PP2AC pathways, proven by transcriptome analysis. Furthermore, 20 reported maize GMS genes were altered in expression levels in *ms33-6038* anthers. The excessive oxidative stress and the metabolic reprogramming resulted in severe phenotypic deficiencies in *ms33-6038* anthers. These findings enrich our understanding of the molecular mechanisms by which ROS and metabolic homeostasis impair anther and pollen development in plants.

## 1. Introduction

Genic male sterility (GMS) is a common phenomenon existing in different crops, such as maize [1], rice [2], wheat [3], and so on. It is now widely used in hybrid production and heterosis research [1,4]. Male sterility is closely related to anther development and pollen formation. In general, the anther wall in higher plants consists of four layers: epidermis, endothecium (En), middle layer, and tapetum from outer to inner. Their formation and functions are precisely regulated during anther development to ensure mature pollen formation [5,6]. So far, hundreds of GMS mutants have been identified in plants [7]. Until now, 39 GMS genes have been identified in maize [1,8,9,10,11], including 21 genes encoding transcriptional factors (TFs), such as ZmMS7 [12,13], ZmOCL4 [14], and ZmMS23 [15]; 13 lipid metabolic genes, such as *ZmMs26* [16,17], *ZmMs30* [18], and *ZmMs33* [19,20,21]; 2 sugar metabolic genes, *ZmMs8* [22] and *ZmMs39* [23]; and 3 genes with other functions. Although the molecular mechanisms of some GMS genes have been well investigated, the physiological and metabolic mechanisms of these genes in regulating male fertility are largely unknown.

Reactive oxygen species (ROS) represent one class of oxygen-containing substances, such as singlet oxygen (^1^O_2_), superoxide anions (O_2_^−^), hydroxyl radicals (•OH), hydrogen peroxide (H_2_O_2_), and nitric oxide (NO) [24]. It is well-known that ROS is an essential regulator of many metabolic pathways, and it plays a critical role in signal transduction [25,26]. During plant anther development, dynamic ROS levels are related to the initiation and progression of tapetal programmed cell death (PCD) [2,27]. For example, in rice, DEFECTIVE TAPETUM CELL DEATH 1 (DTC1) regulates male reproduction through tapetal PCD by modulating ROS-scavenging activity [28]. Besides the *DTC1* gene, many other genes, such as *ARGONAUTE* *2* (*AGO2*) [25], *HEXOKINASE1* (*HXK1*) [25], and a MADS-box transcription factor gene (*MADS3*) [26], are found to regulate male sterility through modulating the ROS accumulation in anthers. In *Arabidopsis*, the stage-specific expressed NADPH oxidase gene (*RBOH*) can affect the timing of tapetal PCD [29]. Although these findings suggest that ROS is involved in anther development and pollen formation, some problems remain, e.g., how ROS homeostasis is maintained during anther development and how ROS status leads to male sterility.

Autophagy is another important physiological process related to metabolic reprogramming, and it plays a critical role in many biological processes, including plant male reproduction [20]. In rice, the autophagy defective mutants, *Osatg7-1* and *Osatg9*, show sporophytic male sterility and fail to undergo anther dehiscence under normal growth conditions [30]. In *Picea abies*, autophagy is necessary for the vacuolar cell death of the embryo suspensor [31]. However, how the autophagy and metabolic reprogramming affect anther development and pollen formation is rarely investigated.

Our previous studies showed that the *ZmMs33* gene is preferentially expressed at anther stages S5 and S6, and it encodes a glycerol-3-phosphate acyltransferase (GPAT) enzyme that catalyzes the first step of glycerophospholipid biosynthesis. Its loss-function mutants exhibited severe male-sterility phenotypes [19,20,32]. While there are still some questions to be answered, i.e., why the mutant anthers showed the severe male-sterility phenotypes and why the appeared stages of mutational phenotypes greatly lagged in time behind the expression stages of the causal mutation gene. Here, a comparative transcriptome analysis combined with cytological and physiological experiments were used to investigate these problems. Excessive ROS content and severely oxidative stress as well as transcriptional alterations of a lot of genes involved in metabolic reprogramming and male reproduction were identified in *ms33-6038* anthers, which may result in the severe phenotypic defects of *ms33-6038* anthers. Furthermore, the comparative transcriptome analysis revealed specific functions of the *ZmMs33* gene in impairing the expression of ROS-related genes. These findings deepen our understanding of molecular mechanisms underlying that ROS and metabolic homeostasis impair anther development in plants.

## 2. Materials and Methods

### 2.1. Plant Materials, Growth Condition, and Phenotypic Characterization

The *ms33-6038* (No. 228I) mutant was initially obtained from the Maize Genetics Cooperation Stock Center (http://maizecoop.cropsci.uiuc.edu, accessed on 20 July 2014). All plant materials were grown at the experimental station of USTB in Beijing. Photos of WT and *ms33-6038* tassels were taken by a Canon EOS 700D camera (Canon, Tokyo, Japan). Fresh anthers of WT and *ms33-6038* at stages S8b and S9 were photographed using an SZX2-ILLB stereomicroscope (Olympus, Tokyo, Japan). Pollen grains were stained with 1% I_2_-KI and photographed using a BX-53F microscope (Olympus, Japan).

### 2.2. Cytological Observation and Microscopy

For transverse section and scanning electron microscope (SEM) analyses, fresh anthers of WT and *ms33-6038* at different developmental stages were fixed in an FAA solution (SL16220, Coolaber, Beijing, China). The subsequent procedures were performed as previously described [33,34]. The semi-thin sections were observed and photographed using a BX-53F microscope (Olympus, Japan). The gold-coated anthers were observed and photographed using a HITACHI S-3400N scanning electron microscope (Hitachi, Tokyo, Japan). The transmission electron microscope (TEM) analysis was performed as previously described [34] in which the fresh anthers were prefixed using a 3% glutaraldehyde solution (G5882, Sigma-Aldrich, City of Saint Louis, MO, USA). The ultrathin sections were observed and photographed using a HITACHI H-7500 transmission electron microscope (Hitachi, Japan).

### 2.3. Transcriptomic Analysis

Transcriptomic data were analyzed as previously described [35]. Specifically, TopHat2 was used to map the clean reads to the maize B73_AGPv4 reference genome with default parameters [36]. Expression levels of coding genes were estimated by the Rsubread package [37]. Anther transcriptomes of *ocl4*, *mac1*, *ms23*, *ms30-6028*, and *p5126-ZmMs7* were reanalyzed by the same method (Table S1). Differentially expressed genes (DEGs) were identified by the edgeR package [38], with a false discovery rate <0.05 and a fold change >3 for each gene. GO enrichment analysis was performed using agriGO [39]. Pathway enrichment analysis was conducted using data from the KEGG database (https://www.kegg.jp/, accessed on 25 March 2019).

### 2.4. Measurement of ROS and •OH

For ROS measurement, H_2_DCF-DA (D6883, Sigma-Aldrich, USA) was dissolved in DMSO (D2650, Sigma-Aldrich, USA) to prepare a 10 mM stock solution. Then, it was diluted to 5 μM as a working solution in 1× HHBS Buffer (MS3510, Maokang Bio-Technology, Shanghai, China). For •OH measurement, HPF (MX4805, Maokang Bio-Technology, China) was dissolved in DMF (227056, Sigma-Aldrich, USA) to prepare a 5 mM stock solution. Then, it was diluted to 10 μM as a working solution in 1× HHBS Buffer (MS3510, Maokang Bio-Technology, China). In the ROS and •OH measurement assays, fresh anthers of WT and *ms33-6038* at different developmental stages were immersed in a 2 mL centrifuge tube, which contained 1 mL of 1× HHBS. After washing the anthers, the supernatant was discarded, a 1 mL working solution (1 mL of H_2_DCF-DA for ROS measurement or 1 mL of HPF working solution for •OH measurement) and 0.5 μL of Silwet L-77 (SL77080596, GE, Boston, MA, USA) was added; it was vacuumed for 15 min and then shaken at 25 °C in darkness (80–100 rmp) for 3 h. After discarding the dye solution, anthers were washed once with 1× HHBS, then 1 mL of 1× HHBS was added into the centrifuge tube [40,41]. The green fluorescence signals were detected by a Leica TCS SP8 confocal microscope (Leica, Wetzlar, Germany). The relative levels of ROS and •OH were quantified by the ImageJ software (http://rsbweb.nih.gov/ij/, accessed on 25 September 2020). Data in a histogram were represented by the average value and the standard deviation of three anthers from a representative of three independent analyses.

### 2.5. Quantitative Real-Time PCR Analysis

Total RNA was isolated from anthers using TRIzol reagent (15596026, Invitrogen, Carlsbad, CA, USA). The concentration and purity of the isolated RNA were measured by a NanoDrop ND-1000 Spectrophotometer (Thermo Fisher, Waltham, MA, USA). After removing genomic DNA with Dnase I (M6101, Promega, Madison, WI, USA), cDNA was synthesized using 5× All-In-One RT MasterMix (G592, abm, Vancouver, BC, Canada). The quantitative real-time PCR (qPCR) analysis was conducted with the corresponding primer set (Table S2) on a QuantStudio 5 Real-Time PCR system (ABI, Waltham, MA, USA), using TB Green Premix EX Tag (RR420A, Takara, Osaka, Japan); *ZmActin1* was used as the internal control. Each sample had 3 biological replicates with 3 technical replicates, the amplification data were analyzed by the 2^−^^△△^^Ct^ method, and the quantitative results were given as means ± standard deviations.

## 3. Results

### 3.1. Transcriptomic Analysis of Wild Type and ms33-6038 Mutant Anthers with Severe Male-sterility Phenotypes Induced by Loss-Function of ZmMs33 Gene

**Figure 1:**
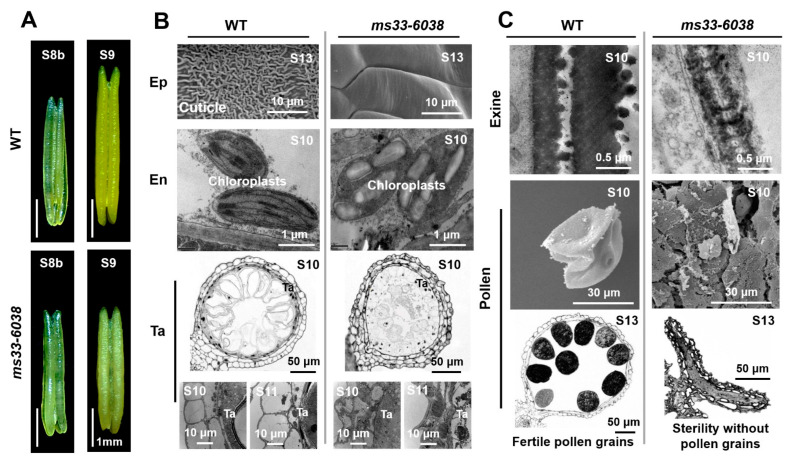
Phenotypic comparisons of anthers, anther wall layers, and pollen grains between WT and *ms33-6038* mutant. (**A**) Phenotypic comparison of WT and *ms33-6038* anthers at stages S8b and S9. (**B**) Phenotypic comparisons of epidermis (Ep), endothecium (En), and tapetum (Ta) layers in WT and *ms33-6038* anthers. (**C**) Phenotypic comparison of WT and *ms33-6038* pollen grains.

The causal mutation gene (*ZmMs33*/*ZmGPAT6*) has been identified for the *ms33-6038* mutant that exhibits severe male-sterility phenotypes previously comprehensively characterized [19,20,21]. Specifically, compared with wild type (WT) anthers, loss of the *ZmMs33* function disrupted anther elongation since stage S9 (Figure 1A). For anther wall layers, unlike the WT anthers that possessed a reticulated accumulation of wax and cutin on the epidermal outer surface at stage S13, invisible starch granules in En chloroplasts at stage S10, and degraded and thin tapetal layers at stages S10 and S11, the *ms33-6038* anthers displayed a smooth outer surface of the epidermis, an excessive starch accumulation in En chloroplasts, and enlarged tapetal cells at the corresponding stages, respectively (Figure 1B). Inside the anthers, the WT pollen possessed typical exine layers, obviously swelled at stage 10, and exhibited round shapes filled with starch granules at stage 13. While, the *ms33-6038* pollen displayed smaller sizes with obviously thin exine layers and failed to develop to mature pollen grains at the corresponding stages, respectively (Figure 1C). These results indicate that loss of the *ZmMs33* function induces severe phenotypic deficiencies of both anther wall layers and pollen grains, at least from stages S9 to S13.

**Figure 2:**
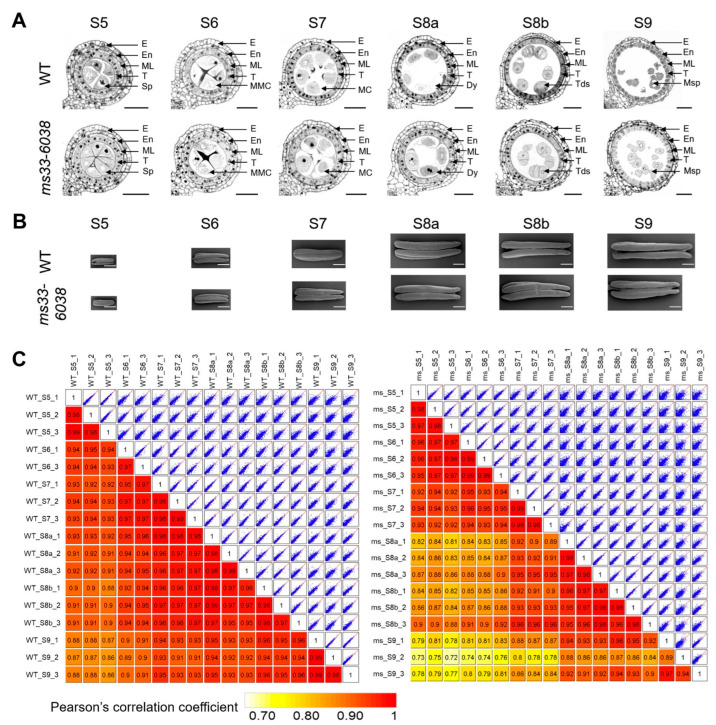
Sampling and the quality of RNA-seq data of WT and *ms33-6038* anther transcriptomes. (**A**) Semi-thin transverse section analysis of WT and *ms33-6038* anthers from stages S5 to S9. Dy, dyad; E, epidermis; En, endothecium; MC, meiocyte; ML, middle layer; MMC, microspore mother cell; Msp, microspore; Sp, sporogenous cell; T, tapetum; Tds, tetrads. Scale bars, 50 μm. (**B**) SEM analysis of WT and *ms33-6038* anthers from stages 5 to 9. Scale bars, 0.5 mm (**C**) Comparisons of the transcriptomic data of WT (**left**) and *ms33-6038* (**right**) anthers at six stages. The similarity between two samples was estimated by Pearson’s correlation coefficient.

To investigate possible mechanisms underlying the severe effects of *ZmMs33* deficiency on anther development, we performed a transcriptome analysis. Considering the *ZmMs33* gene is highly expressed in anthers at stages S5 and S6 [20] and the phenotypic changes obviously appeared since stage S9, the *ms33-6038* and WT anthers of six stages from S5 to S9 were included in RNA-sequencing (RNA-seq) analysis. It is worth mentioning that the developmental stages of anther samples were not only estimated by the anther lengths but also determined by the cytological evidence from the semi-thin transverse section analysis of maize anthers from the same spikelet of sampled anthers (Figure 2A,B). Three biological replicates were conducted in both WT and *ms33-6038* anthers at each of the six stages. The results of similarity analysis suggested a high repeatability among 3 biological replicates (Figure 2C and Figure S1), indicating that the transcriptomes have a good representation of transcriptional changes in both WT and *ms33-6038* anthers from stages S5 to S9.

**Figure 3:**
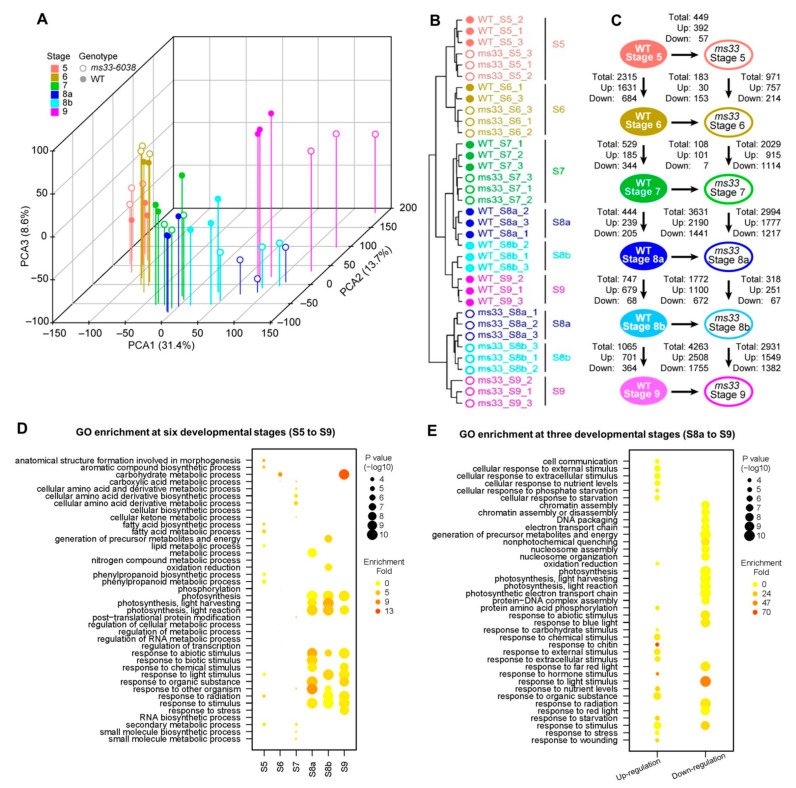
Transcriptome analysis of RNA-seq data of WT and *ms33-6038* anthers. (**A**) Principal component analysis of WT and *ms33-6038* anther transcriptomes from stages S5 to S9. (**B**) Hierarchical clustering of WT and *ms33-6038* anther transcriptomes from stages S5 to S9. (**C**) The number of DEGs between WT and *ms33-6038* anther transcriptomes and between adjacent developmental stages from stages S5 to S9. (**D**) GO enrichment analysis of DEGs between WT and *ms33-6038* anther transcriptomes at each of six stages. (**E**) GO enrichment analysis of up- or down-regulated DEGs in *ms33-6038* anther transcriptome compared to WT from stages S8a to S9.

Principal component analysis (PCA) of RNA-seq data revealed that *ms33-6038* or WT anther samples at the same stages were clustered together and the anther samples were successively arranged with the developmental stages identified by the cytological evidence, emphasizing that the transcriptomes are usable (Figure 3A). Hierarchical clustering analysis of the transcriptomes showed that samples from stages S5 to S7 were clustered by the three developmental stages, while samples from stages S8a to 9 were clustered according to the genotypes of WT or *ms33-6038* (Figure 3B). Moreover, the numbers of DEGs between *ms33-6038* and WT anthers at stages S5, S6, and S7 do not exceed the numbers of DEGs between stages S6 and S5, stages S7 and S6, and stages S8a and S7, respectively (Figure 3C). In contrast, the numbers of DEGs between *ms33-6038* and WT anthers from stages S8a to S9 obviously exceeded those between the adjacent stages in WT anthers (Figure 3C). These results indicate that *ZmMs33* deficiency has a relatively small effect on the anther transcriptomes before microsporocyte meiosis (stages S5, S6, and S7), while dramatic changes occur at anther meiotic (stages S8a and S8b) and postmeiotic (stage S9) processes. Gene ontology (GO) analysis suggested that the DEGs between *ms33-6038* and WT anthers from stages S5 to S7 were functionally enriched in dispersed biological processes, including fatty acid (FA) and phenylpropanoid metabolisms and stimulus responses at stage S5, carbohydrate metabolism at stage S6, and cellular amino acid derivate metabolism at stage S7 (Figure 3D). However, the DEGs from stages S8a to S9 were significantly enriched in consistent biological processes, including photosynthesis and stress responses (Figure 3D). Specifically, from stages S8a to S9, the upregulated DEGs were annotated to be involved in stress responses and the downregulated DEGs were functionally enriched in photosynthesis and electron transport chain related to carbon and energy metabolisms (Figure 3E). Taken together, the *ZmMs33* deficiency leads to abnormally activated stress responses and reprogrammed metabolisms at the transcriptional level, which may result in severe phenotypic defects in anther development.

### 3.2. ZmMs33 Deficiency Caused Severe Oxidative Stress in Maize Anthers

**Figure 4:**
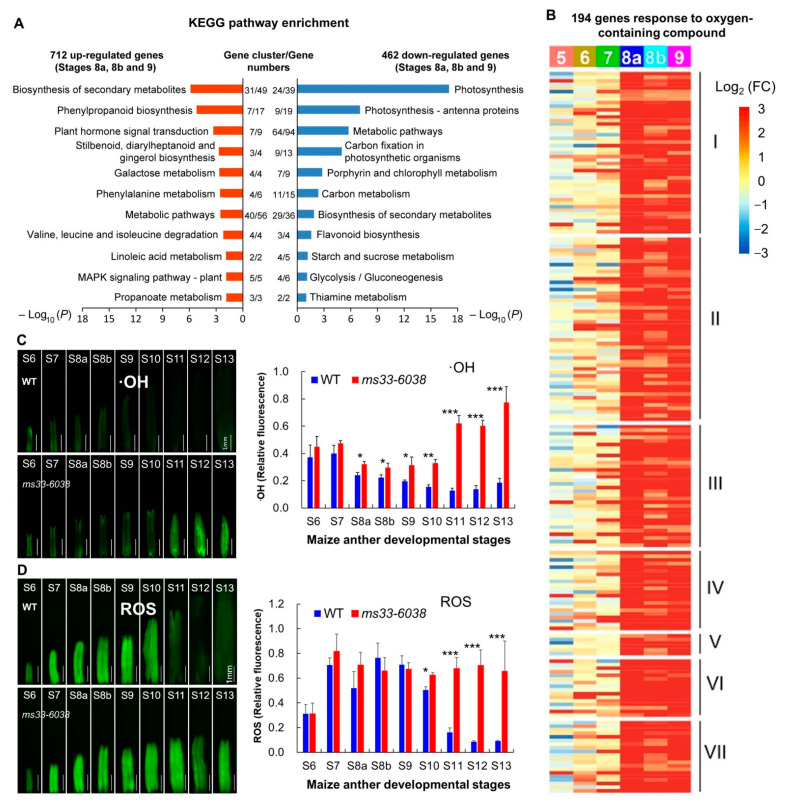
Active oxidative stress responses and increased ROS accumulation in *ms33-6038* anthers. (**A**) KEGG pathway enrichment analysis of 712 upregulated and 462 downregulated genes between *ms33-6038* and WT anthers from stages S8a to S9. (**B**) Expression changes of 194 genes related to the response to oxygen-containing component between *ms33-6038* and WT anthers from stages S5 to S9. The 194 genes were functionally grouped into 7 types including ROS response (Table S3). FC, fold change. (**C**,**D**) Fluorescence imaging and relative quantification of the •OH contents (**C**) and the total ROS levels (**D**) in WT and *ms33-6038* anthers from stages S6 to S13. * *p* < 0.05, ** *p* < 0.01, *** *p* < 0.001, Student’s *t*-test.

To reveal which type of stress is mainly induced in *ms33-6038* anthers, we conducted KEGG pathway enrichment analysis and found that genes involved in plant MAPK signaling pathway and plant hormone signal transduction were activated in *ms33-6038* anthers, while the downregulated genes mainly functioned in the primary metabolic pathways (Figure 4A). The MAPK signaling pathway was reported to respond to various environmental stresses, such as hyperosmolarity, extreme pH, temperature changes, and ROS as one type of plant defense signal [42]. ROS homeostasis is of great importance for the development of germ cells and tapetal cells in plant anthers [29,43]. Since *ms33-6038* anthers were not stimulated through external stresses, it can be speculated that the severe phenotypic deficiencies in *ms33-6038* anthers may be endogenously induced by oxidative stress. To test it, we investigated the expression changes of genes included in the GO term of the response to the oxygen-contained compound (GO term ID: 1901700) and found that 194 related genes were obviously upregulated in *ms33-6038* anthers mainly from stages S8a to S9 (Figure 4B and Table S3). Furthermore, the levels of •OH, an important ROS type formed non-enzymatically by the Fenton reaction between H_2_O_2_ and Fe^2+^, were significantly higher in *ms33-6038* anthers from stage S8a to S13 compared with those of WT anthers (Figure 4C). Besides, the total ROS levels in *ms33-6038* anthers were significantly higher than those of WT anthers from stages S10 to S13 (Figure 4D). These findings suggest *ZmMs33* deficiency induces the excessive ROS levels in anthers and activates a large number of genes in response to oxidative stress.

**Figure 5:**
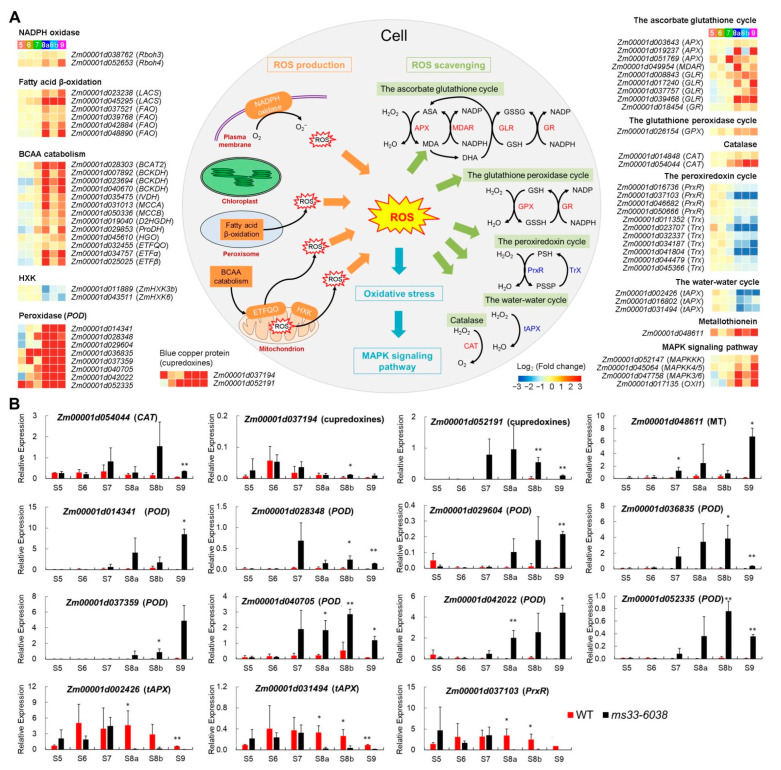
Transcriptional changes of genes related to ROS metabolism and oxidative stress responses in *ms33-6038* anthers. (**A**) Expression changes of 64 genes involved in ROS production, ROS scavenging, and oxidative stress responses. (**B**) Validation of expression change patterns of 15 representative genes related to ROS metabolism and oxidative stress response by qPCR analysis. * *p* < 0.05, ** *p* < 0.01, Student’s *t*-test.

To further reveal the molecular mechanism underlying the excessive ROS accumulation in *ms33-6038* anthers, we comprehensively investigated expression changes of 64 genes involved in ROS generation (4 pathways), scavenging (5 pathways), and signaling (the MAPK pathway), according to previous studies (Figure 5A and Table S4). In *ms33-6038* anther transcriptomes, 2 genes encoding NADPH oxidases, 6 genes involved in FA β-oxidation, and 13 genes in branched-chain amino acid (BCAA) catabolism pathway were upregulated mainly from stages S8a to S9, while 2 genes encoding mitochondria-located HXKs were downregulated, which could promote ROS generation (Figure 5A). In addition, 11 genes involved in the peroxiredoxin cycle and 3 in water–water cycle were downregulated mainly from stages S8a to S9, contributing to a reduced ability of ROS scavenging in *ms33-6038* anthers (Figure 5A). However, 10 genes participated in ascorbate glutathione and glutathione peroxidase cycles, 2 catalase genes, 8 peroxidase genes, 2 blue copper protein genes, and 1 metallothionein gene, were upregulated in *ms33-6038* anthers (Figure 5A), which may be induced by the high ROS levels. Furthermore, four genes involved in the MAPK signaling pathway were transcriptionally activated in *ms33-6038* anthers (Figure 5A). The expression patterns of 15 representative genes were confirmed by qPCR analysis (Figure 5B). These results indicate that the excessive ROS content in *ms33-6038* anthers is produced most probably via the comprehensive activation of ROS generation pathways yet the repression of ROS scavenging pathways, which together induce the extreme oxidative stress and activate oxidative stress responses by the MAPK signaling pathway.

### 3.3. The Uniqueness of ZmMs33 Deficiency-Induced Excessive Oxidative Stress among Maize GMS Mutants

**Figure 6:**
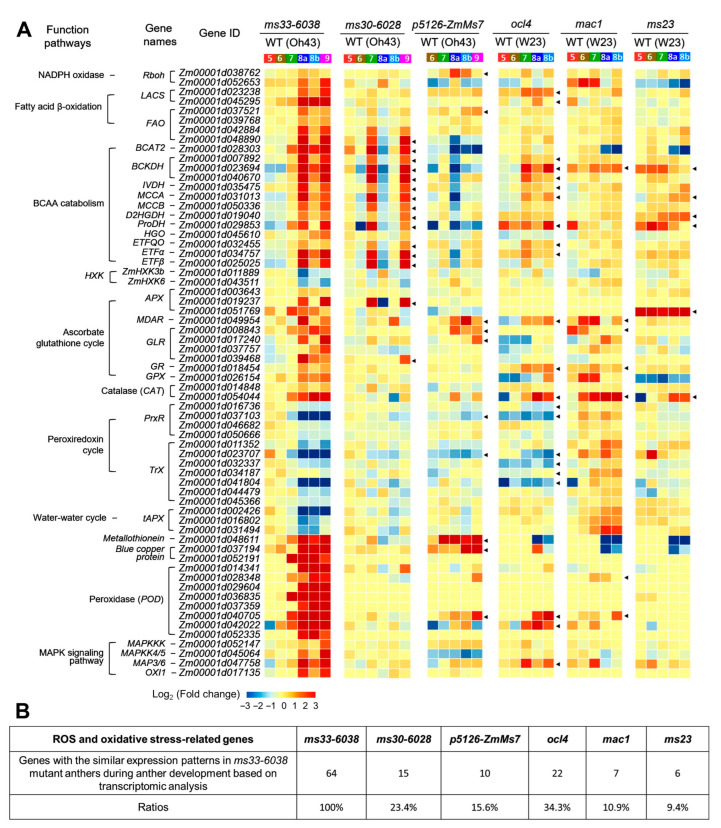
Transcriptome analysis of ROS and oxidative stress-related genes between *ms33-6038* and each of the other five GMS lines. (**A**) The expression heatmap of ROS and oxidative stress-related genes in *ms33-6038* and the other five GMS lines (*ms30-6028*, *p5126-ZmMs7*, *ocl4*, *mac1*, and *ms23*). Arrow indicates a gene with a similar expression change to that in *ms33-6038* anthers. (**B**) A summary of the numbers and percentages of ROS and oxidative stress-related genes with similar expression changes to *ms33-6038* in each GMS line.

The excessive oxidative stress can impact the differentiation of sporogenous cells and the degeneration of tapetal cells in anther development [25,29,43]. The *ocl4*, *mac1*, and *ms23* GMS lines displayed abnormal differentiations of anther wall layers and sporogenous cells [14,44,45] (Figure S2). Both *ZmMs30* deficiency (*ms30-6028*) and *ZmMs7* overexpression (*p5126-ZmMs7*) anthers exhibited abnormal tapetum development [13,18]. All of these five GMS lines exhibit oxidative stress-induced phenotypic deficiencies during anther development. To examine whether *ms33-6038* and other GMS lines share a similar mechanism underlying the abnormal ROS content that impair anther development and male fertility, we performed a comparative transcriptome analysis on the 64 genes related to ROS metabolism and oxidative stress responses between *ms33-6038* and each of the 5 GMS lines (*ms30-6028*, *p5126-ZmMs7*, *ocl4*, *mac1*, and *ms23*). Unlike *ms33-6038* anthers displaying a comprehensive transcriptional reprograming of the 64 maker genes, only a few of these genes in the 5 investigated GMS lines have changed expression patterns similar with those of *ms33-6038* anthers (Figure 6). Specifically, the *p5126-ZmMs7*, *mac1*, and *ms23* anthers possessed fewer genes (10/64, 7/64, and 6/64, respectively), with similar expression changes to those of *ms33-6038* anthers, and these genes were separately distributed in several pathways. In the MAPK signaling pathway, only one maker gene was upregulated in *ocl4* anthers. These results revealed that the comprehensive transcriptional changes of ROS metabolic and signaling genes in *ms33-6038* anthers were not observed in the five investigated GMS lines, indicating that the excessive oxidative stress and the expression alterations of anti-oxidative genes were specific in *ms33-6038* anthers.

### 3.4. ZmMs33 Deficiency-Induced Oxidative Stress Activated Premature Autophagy and Metabolic Reprogramming in Tapetum, Leading to Transcriptional Changes of Other GMS Genes

**Figure 7:**
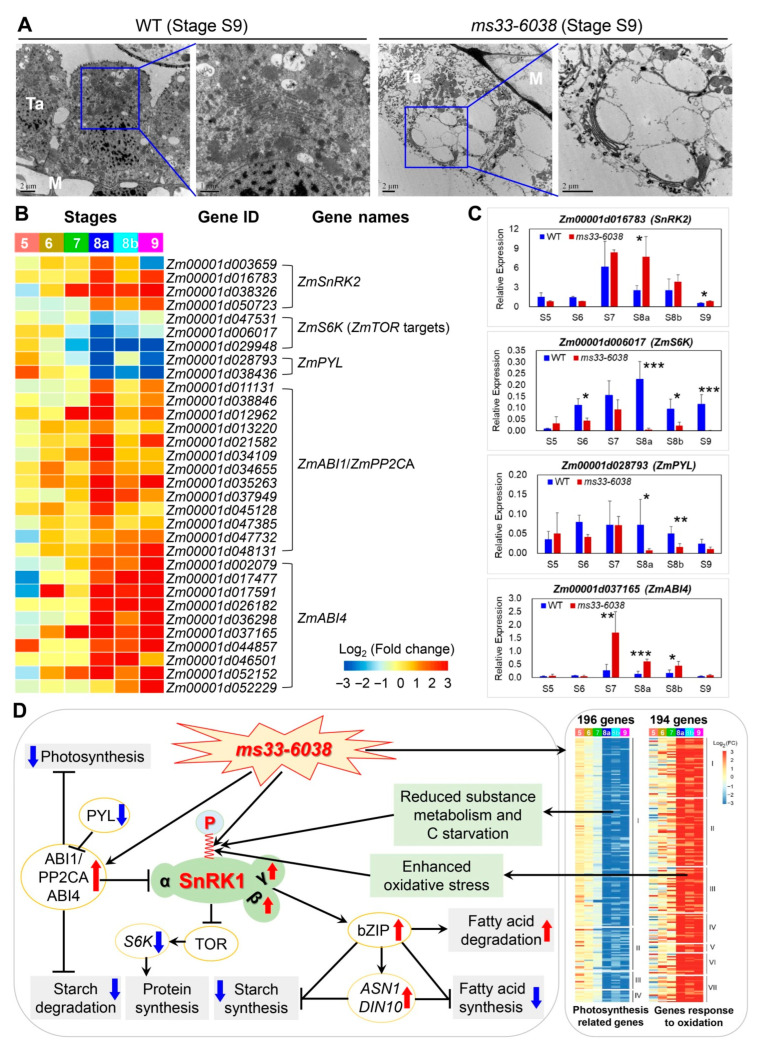
Premature and excessive tapetal autophagy and the underlying mechanism of metabolic reprogramming in *ms33-6038* anthers. (**A**) Premature and excessive tapetal autophagy in *ms33-6038* anthers. (**B**) Expressions changes of genes involved in metabolic reprogramming related to SnRK1, TOR, PP2CA pathways, and oxidative stress responses in the *ms33-6038* anther transcriptomes. (**C**) Validation of expression change patterns of four representative genes related to metabolic reprogramming by qPCR analysis. * *p* < 0.05, ** *p* < 0.01, *** *p* < 0.001, Student’s *t*-test. (**D**) The potential molecular mechanism of metabolic reprogramming in *ms33-6038* anthers.

Tapetum degeneration and autophagy usually occur at anther developmental stage S10 in maize, which is triggered by a transient oxidative burst in tapetum [20] and is closely associated with cellular vacuolations [46]. Here, we found the tapetal cells of *ms33-6038* anthers experienced severe vacuolations and even lost their nuclear structure compared with those of WT anthers at stage S9 by TEM analysis (Figure 7A). The premature and excessive vacuolation have been demonstrated to be mediated by the activated SnRK1-bZIP signaling and the downstream genes (*ASN1* and *DIN10*) [47], which induced metabolic reprogramming by activating catabolism and repressing anabolism in *ms33-6038* anthers [20]. Specifically, in *ms33-6038* anther transcriptomes, the metabolic reprogramming was proven by the transcriptional alterations of genes involved in primary metabolic pathways from stages S8a to S9 (Figures S3–S5). To further uncover molecular mechanisms underlying the excessive autophagy and metabolic reprogramming besides the active SnRK1-mediated sugar signaling pathway previously reported in *ms33-6038* anthers [20], we investigated the expression changes of genes in controlling metabolic homeostasis, including the TOR signaling pathway, PYL-PP2CA pathway, and SnRK2-mediated stress response pathway (Table S5). In *ms33-6038* anthers, we found the repressed TOR (an energy and nutrient sensor) reflected by the downregulated expression of *S6K* gene involved in protein synthesis [48,49] (Figure 7B). In addition, four *PP2CA* genes, encoding core regulators negatively controlling plant photosynthesis and starch degeneration through abscisic acid (ABA) signaling, were upregulated in *ms33-6038* anthers, consistent with the downregulated expression of two *PYL* genes, encoding ABA receptors those inhibit the phosphorylation activities of PP2CA proteins [50,51,52] (Figure 7B). Meanwhile, three *SnRK2* homologous genes responsible for abiotic stress responses were upregulated in *ms33-6038* anthers (Figure 7B). The qPCR results confirmed the expression changes of four representative genes (Figure 7C).

Collectively, the excessive ROS accumulation and oxidative stress in *ms33-6038* anthers induce severe autophagy and metabolic reprogramming and the activated SnRK1-bZIP signaling pathway that can repress starch and FA syntheses, following interrupted protein synthesis by inhibiting TOR activity, repressed photosynthesis and starch degeneration by the activated PP2AC pathway and additionally activated SnRK2-mediated stress response (Figure 7D), finally result in severe phenotypic deficiencies of anther wall layers and pollen grains.

**Figure 8:**
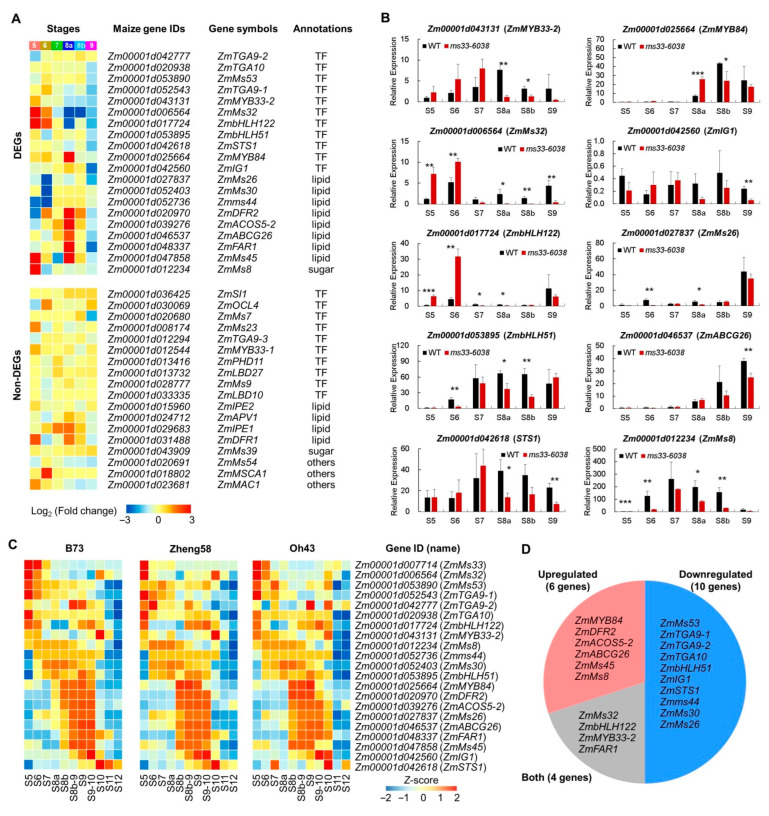
Expression changes of all reported maize GMS genes in *ms33-6038* anthers. (**A**) Expression changes of all 38 reported maize GMS genes in *ms33-6038* anther transcriptomes. (**B**) Validation of the expression change patterns of 10 differentially expressed GMS genes by qPCR analysis. * *p* < 0.05, ** *p* < 0.01, *** *p* < 0.001, Student’s *t*-test. (**C**) Expression patterns of *ZmMs33* and 20 differentially expressed GMS genes in anther transcriptomes of 3 maize lines. (**D**) Numbers of upregulated and downregulated genes among the 20 differentially expressed GMS genes.

To investigate whether *ZmMs33* deficiency alters the expression patterns of other GMS genes and to further reveal the underlying molecular mechanism of the severe phenotypic defects, we investigated transcriptional changes of all reported maize GMS genes in the *ms33-6038* anther transcriptomes. Among the 38 reported maize GMS genes except *ZmMs33* (Table S6), 20 of them (11 TF genes, 8 lipid metabolic genes, and 1 sugar metabolic gene) were significantly altered in expression levels in the *ms33-6038* anther transcriptomes (Figure 8A) from stages S5 to S9, 10 of which were confirmed by qPCR analysis (Figure 8B). These results suggest loss of the *ZmMs33* function severely impairs the expression of half of the reported maize GMS genes. *ZmMs33* was highly expressed at stages S5 and S6 [20]. The expression peaks of the 20 GMS genes in the anther transcriptomes of 3 maize lines have consistent patterns ranging from stages S5 to S13 (Figure 8C), suggesting that *ZmMs33* deficiency induces a long-term effect on the transcriptional changes of GMS genes that are expressed after *ZmMs33* gene expression. Half of the 20 GMS genes were downregulated in expression levels in *ms33-6038* anthers during stages S5 to S9, 6 genes were upregulated, and the remaining 4 genes displayed up- or down-regulated expressions at separated stages, indicating a complex effect of *ZmMs33* deficiency on the expressions of other GMS genes. Taken together, loss of the *ZmMs33* function induces a severe, long-term, and complex transcriptional reprogramming of a large number of the reported maize GMS genes, which may contribute to the excessive phenotypic alterations in *ms33-6038* anthers.

**Figure 9:**
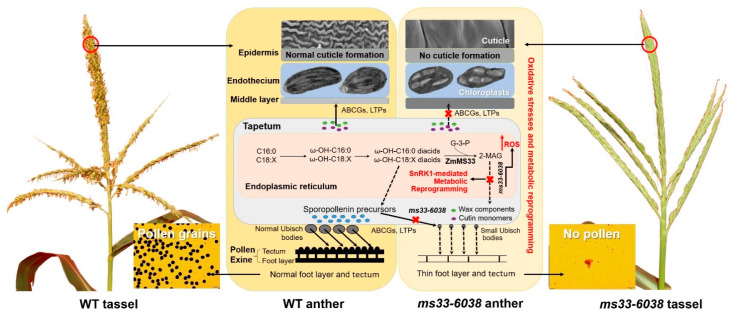
A reconstructed working mode underlying excessive phenotypic deficiencies in *ms33-6038* anthers. *ZmMs33* deficiency induces an excessive ROS accumulation and metabolic reprogramming in tapetum, further impairs the expression and function of many other GMS genes, and finally leads to severe phenotypic deficiencies in the development of an anther wall and pollen grains.

Collectively, based on results previously reported and obtained in this study, we proposed a working model underlying the excessive phenotypic deficiencies in *ms33-6038* anthers (Figure 9). Specifically, loss of the *ZmMs33* function directly disrupts glycerophospholipid metabolism [20], leading to reduced lipid accumulation and transport related to the smooth outer surface of anthers, disabled En chloroplasts, and abnormally thin pollen exine. The specific and excessive ROS accumulation and oxidative stress responses in *ms33-6038* anthers induce the premature and severe tapetal autophagy and metabolic reprogramming associated with the activated SnRK1 [20], PYL-PP2CA and SnRK2 signaling pathways, as well as the inhibited TOR pathway; meanwhile, the expression of many GMS genes that further excessively impaired the development of a four-layer anther wall and microspores were disturbed (Figure 9).

## 4. Discussion

### 4.1. Severe ROS Accumulation and Oxidative Stress in ZmMs33-Deficient Anthers among Maize GMS Lines

*ZmMs33* encodes a GPAT enzyme involved in glycerophospholipid biosynthesis [19,32]. The *ZmMs33*-deficient anthers produce the reduced contents of membrane lipid components, which impair the function of En chloroplasts [20]. Energy-generating processes including photosynthesis produce ROS as by-products [53]. The abnormal membrane structure of En chloroplasts results in the leakage of electron transport chains in the photosynthesis process, increasing the cellular ROS content that in turn impairs the photosynthetic activity [54]. In addition, loss of the *ZmMs33* function leads to enhanced FA degradation or β-oxidation in anthers suffering metabolic reprogramming [20], which is another source of excess ROS content. In *ms33-6038* anthers, a large number of genes involved in four ROS production pathways and five ROS scavenging processes are changed in expression levels, breaking the critical balance of ROS dynamics and inducing excessive ROS content. The high ROS concentration can induce oxidative stress, causing cellular damage of DNA strand breaks, lipid peroxidation, membrane leakage, and cell lysis [55]. It has been proven that the number of germ cells in anthers is determined by their surrounding oxidizing status [43] and that a transient oxidative burst is closely related to tapetal degeneration [27], indicating the redox environment— represented mainly by the ROS content—is a critical determinant in plant anther development and male reproduction. A lot of genes in controlling the ROS dynamics during anther development have been characterized, and their functional deficiencies frequently cause abnormal PCD and degeneration of tapetum [26,28,29]. Similarly, excessive ROS content triggers premature and severe vacuolations in *ms33-6038* tapetal cells, leading to premature tapetal degeneration and abnormal pollen exine, as well as the lack of a cutin and wax layer [20,32]. Unlike *ms33-6038* anthers, the majority of marker genes involved in ROS production and scavenging were normally expressed in the other five investigated GMS lines during anther development. The result that the ROS content seriously exceeds the homeostatic level in *ms33-6038* anthers may reflect the specific role of this *GPAT* gene in maintaining cellular ROS homeostasis by regulating the production of membrane lipids and impairing the homeostasis of FA metabolism among the reported maize GMS genes in controlling anther development and pollen formation.

### 4.2. The Phenotypic Alterations Lag behind the Preferentially Expressed Stages of ZmMs33 during Maize Anther Development

The phenotypic changes of GMS lines usually appear at or closely behind the stages when the investigated GMS gene is preferentially expressed [1,8]. The *ZmMs33* gene is preferentially expressed at stages S5 and S6 before microspore meiosis, while obvious morphologic alteration was not observed in *ms33-6038* anthers at the two stages. The phenotypic differences in *ms33-6038* compared with WT anthers appeared mainly after stage S8a, including the enhanced •OH and ROS levels and premature tapetal vacuolations observed in this study, as well as the arrested anther length and En chloroplast development, the increased H_2_O_2_ level, the metabolic reprogramming, and the abnormal results in transverse section analysis revealed in previous studies [20,21,32]. The phenotypic lagging is reflected by the numbers and functions of DEGs in the *ms33-6038* anther transcriptomes from stages S5 to S9. The numbers of DEGs between *ms33-6038* and WT anthers at stages S5 and S6 are greatly less than the numbers of DEGs required by the normal development of WT anthers from stages S5 to S7, indicating the transcriptional changes in *ms33-6038* anthers at stages S5 and S6 are insufficient to switch the normal development trend of anthers to an abnormal route. However, along with the development of *ms33-6038* anthers, more genes are differentially expressed since stage S8a, exceeding the number of DEGs during normal anther development, which start to induce obvious phenotypic alterations in *ms33-6038* anthers. In addition, the DEGs from stages S5 to S7 are functionally enriched in the biological processes not related to anther development. In contrast, the DEGs from stages S8a to S9 are enriched in the functions of metabolic reprogramming, plant hormone signal transduction, and those stress responses that fundamentally impair anther development [20,56,57]. Besides the lagging of transcriptional changes, metabolic reprogramming can generate energy and carbon supply endogenously to keep anther alive before the microspore meiosis in *ms33-6038* anthers [20]. When microspores undergo meiosis, the consumption of internal substances is insufficient to support the survival of *ms33-6038* anthers in which severe phenotypic alterations appear. Thus, both the transcriptional and metabolic alterations contribute to the lagging of severe phenotypic changes in *ms33-6038* anthers, leading to the specificity of phenotypic lagging in *ms33-6038* anthers among the reported GMS lines.

### 4.3. The Severe Transcriptional Reprogramming Induced by ZmMs33 Deficiency during Maize Anther Development

Though *ZmMs33* encodes an enzyme, its deficiency results in thousands of DEGs in anther transcriptomes from stages S8a to S9. Interestingly, the number of DEGs in *ms33-6038* anthers is equivalent to those in the GMS lines with functionally defective TF-encoding genes, such as *ZmMs23* and *ZmMs7* [13,15,58]. Besides the comprehensive regulatory roles of TFs on target genes, genome-wide transcriptional reprogramming can be triggered by cellular stresses, including oxidative stress [59,60]. By the transcriptional reprogramming, plants can maintain metabolic and physiological homeostasis under adverse growth environments [59]. Thus, the transcriptional reprogramming in *ms33-6038* anthers may be partially triggered by the excessive oxidative stress induced by loss of the *ZmMs33* function. This inference is confirmed by the results of GO and KEGG enrichment analyses in which the functions of DEGs are closely related to abiotic stress responses. Moreover, 11 reported maize GMS genes encoding TFs, including the key regulator genes modulating tapetal development (*ZmbHLH122*, *ZmbHLH51*, and *ZmMs84*) [10,61,62] are changed in transcription levels in *ms33-6038* anthers, which strengthen transcriptional reprogramming by directly impairing the expression of target genes. Thus, *ZmMs33* deficiency disturbs the expression of genes involved in both the stress response and the regulatory network of male reproduction, which brings about genome-wide transcriptional reprogramming. Furthermore, under transcriptional reprogramming, the severe oxidative stress induced by *ZmMs33* deficiency finally causes complete male sterility, prompting us to think about the possible crosstalk between stress response and male sterility during plant anther development in a further study.

## 5. Conclusions

In conclusion, this study shows that loss of the *ZmMs33* function can lead to ROS accumulation and severe oxidative stress in maize anthers, which are mediated by specific underlying mechanisms among the investigated maize GMS lines. Additionally, due to loss of the *ZmMs33* function, anthers suffered metabolic reprogramming mediated by several key regulators (i.e., TOR, PP2CA, and SnRK2) in controlling metabolic homeostasis and the stress response. Excessive oxidative stress and metabolic reprogramming may induce the transcriptional reprograming of tens of reported maize GMS genes, which could explain the severe phenotypic changes in *ZmMs33*-deficient anthers. The interplay among lipid metabolism, ROS metabolism, and plant male sterility are worthy of further study.

## Data Availability

All data included in this study are available upon request by contacting the corresponding author.

## Supplementary Materials

The following supporting information can be downloaded at: https://www.mdpi.com/article/10.3390/cells11152318/s1, Table S1: A summary of the transcriptomic data used in this study; Table S2: Primers used in this study; Table S3: A list of 194 genes related to the response to oxygen-containing components; Table S4: A list of 64 genes involved in ROS production, ROS scavenging, and oxidative stress responses; Table S5: A list of 32 genes involved in metabolic reprogramming; Table S6: A list of 39 reported maize GMS genes; Figure S1: Similarities of anther transcriptomic data in WT and *ms33-6038* mutant from stages S5 to S9; Figure S2: Phenotypes of anthers and pollen grains of *ms33-6038* and the other five maize GMS lines (*ms30-6028*, *p5126-ZmMs7*, *ocl4*, *ms23*, and *mac1*); Figures S3–S5: Expression changes of genes involved in primary metabolism pathways in the *ms33-6038* anther transcriptomes at stages S5 and S6 (Figure S3), stages S7 and S8a (Figure S4), and stages S8b and S9 (Figure S5).
