# Supplementary material for: The Loss-Function of the Male Sterile Gene ZmMs33/ZmGPAT6 Results in Severely Oxidative Stress and Metabolic Disorder in Maize Anthers
Source: Cells. 2022 Jul 27;11(15):2318. doi: 10.3390/cells11152318 (PMC9367433; doi:10.3390/cells11152318)
Supplement: Supplementary file 1 [file cells-11-02318-s001.zip › Supplemental Figures.pdf]

cells

Supplemental Figures

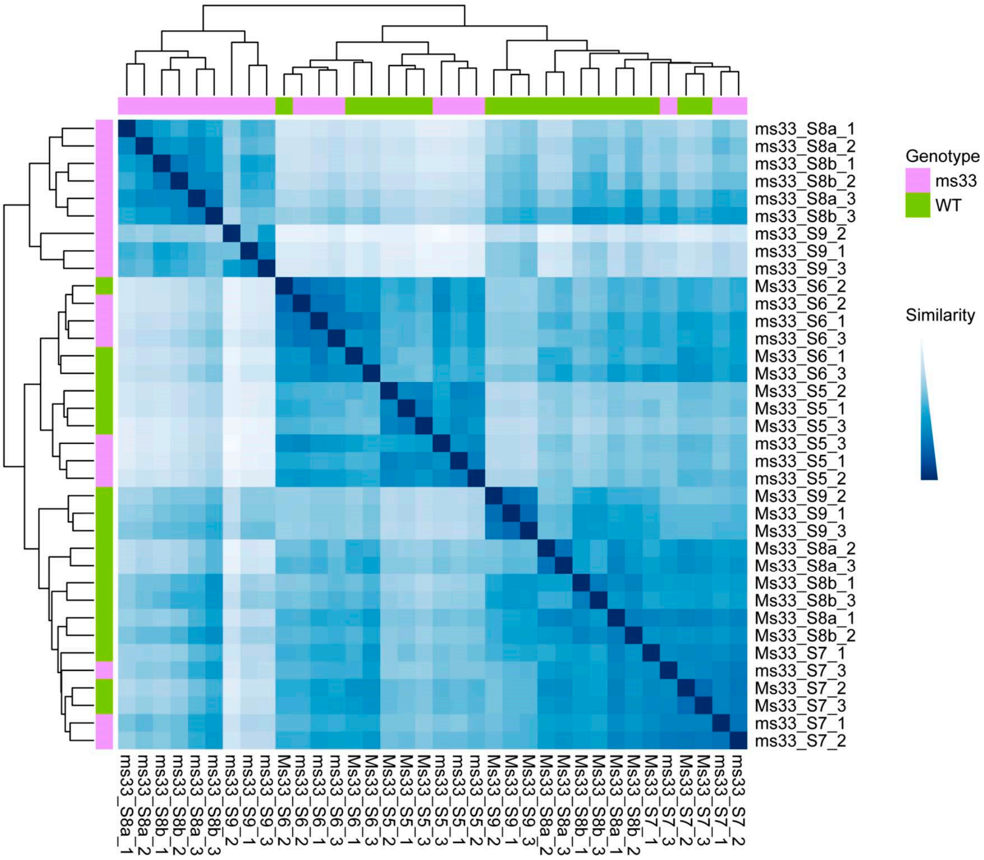

Figure S1. Similarities of anther transcriptomic data in WT and *ms33-6038* mutant from stages S5 to S9.

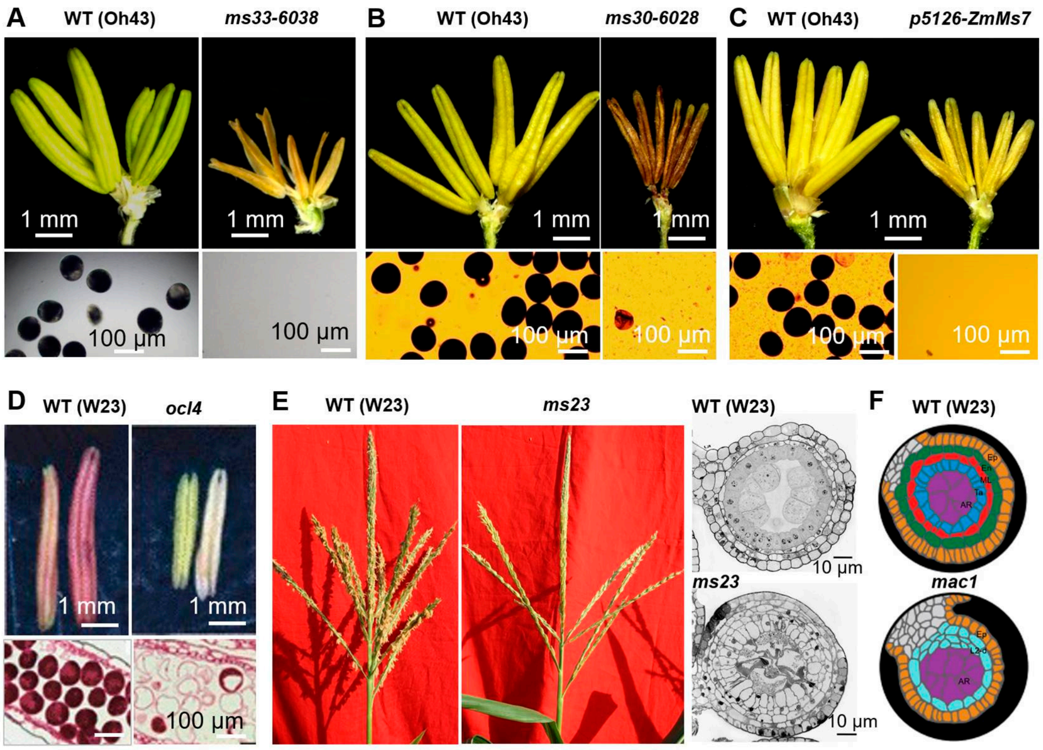

Figure S2. Phenotypes of anthers and pollen grains of *ms33-6038* and the other five maize GMS lines (*ms30-6028*, *p5126-ZmMs7*, *ocl4*, *ms23*, and *mac1*).

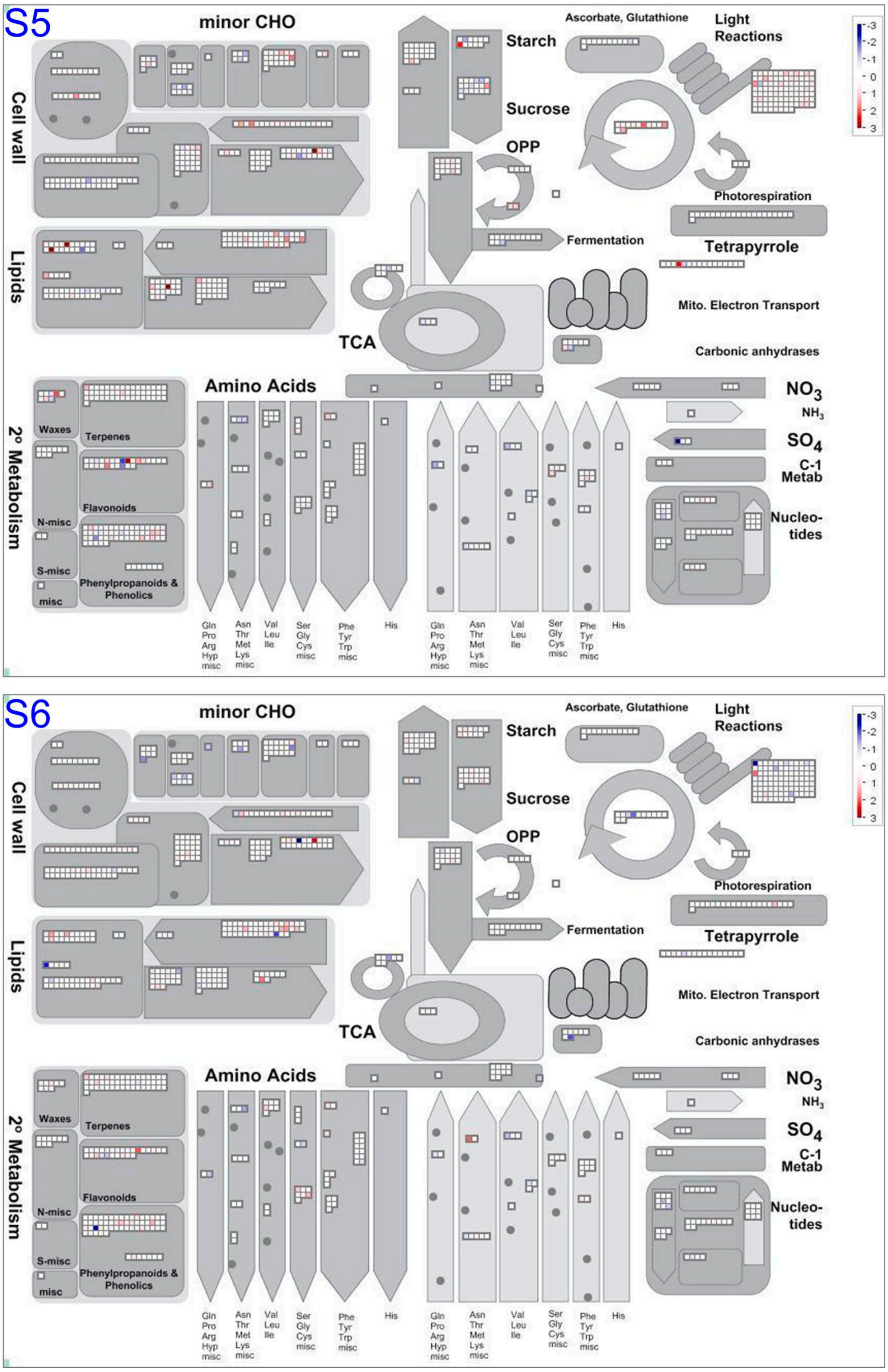

Figure S3. Expression changes of genes involved in primary metabolism pathways in the *ms33-6038* anther transcriptomes at stages S5 and S6.

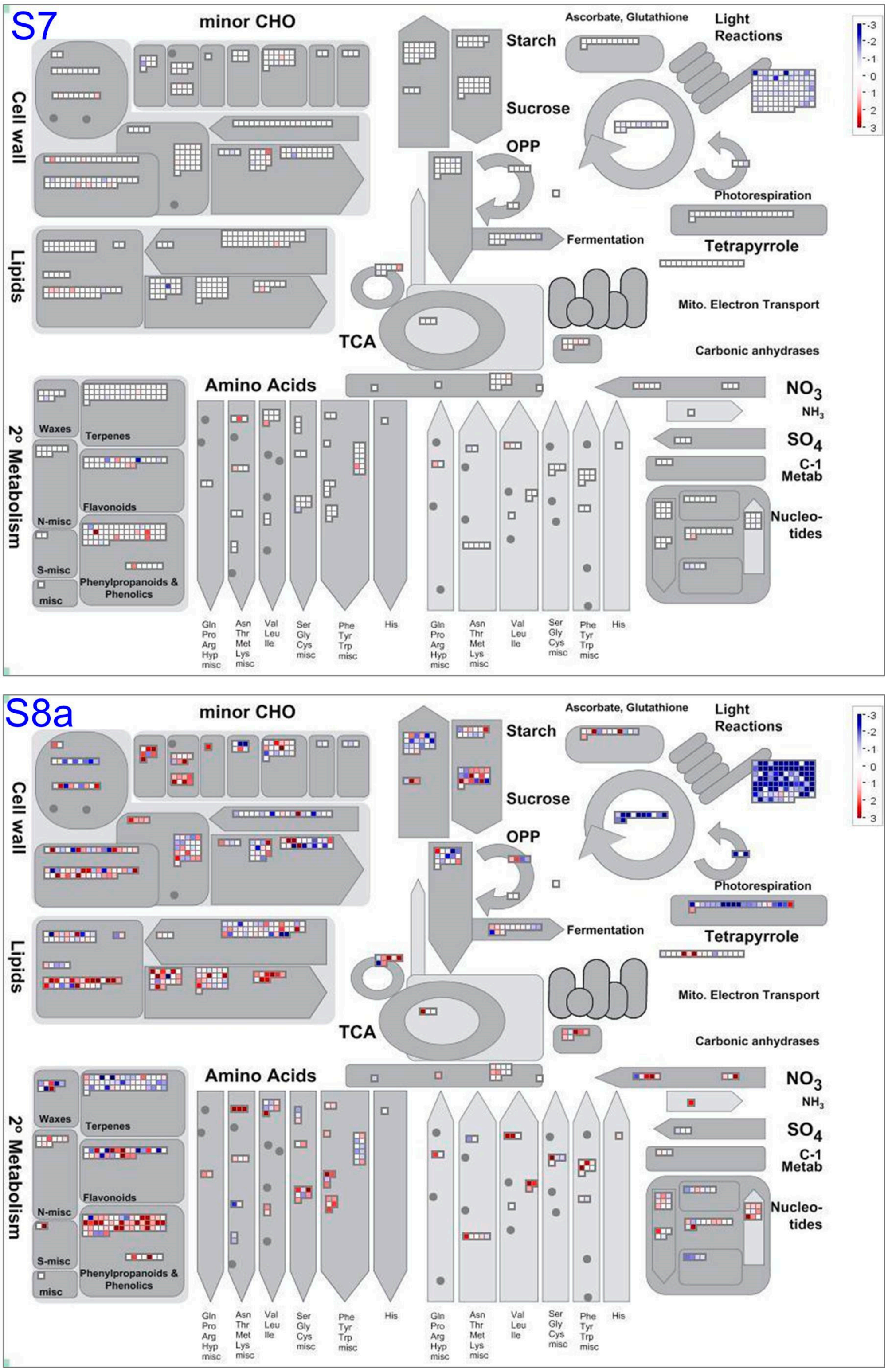

Figure S4. Expression changes of genes involved in primary metabolism pathways in the *ms33-6038* anther transcriptomes at stages S7 and S8a.

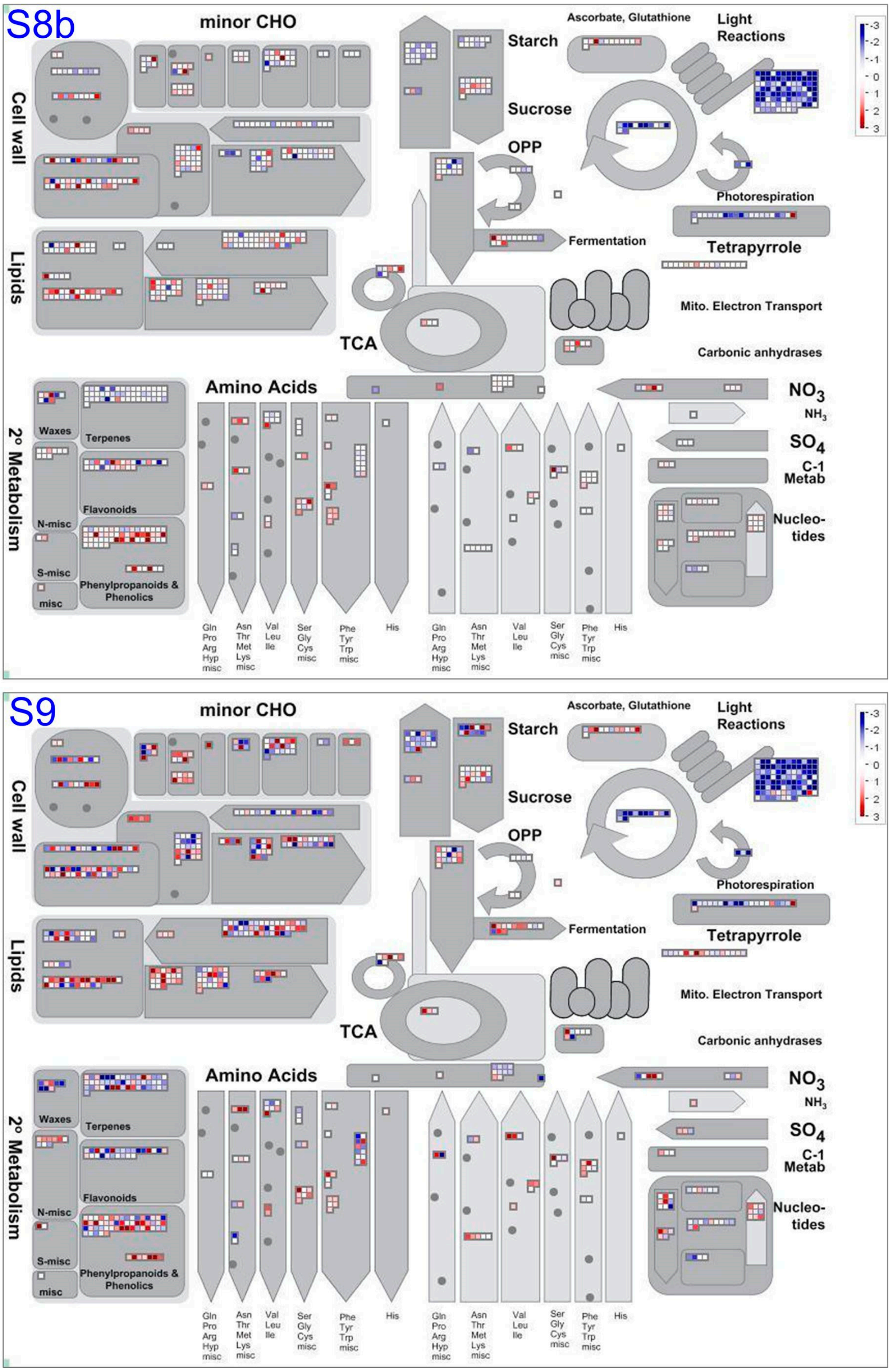

Figure S5. Expression changes of genes involved in primary metabolism pathways in the *ms33-6038* anther transcriptomes at stages S8b and S9.
